# Case–Control Studies and Risk Factors for Cataract in Two Population Studies in Nigeria

**DOI:** 10.4103/0974-9233.71592

**Published:** 2010

**Authors:** S. I. Echebiri, P. G. C. Odeigah, S. N. Myers

**Affiliations:** Department of Cell Biology and Genetics, Faculty of Science, University of Lagos, Akoka Yaba, Lagos, Nigeria; 1 ECWA Eye Hospital, Kano, Nigeria

**Keywords:** Case–Control Studies, Cataract, Fulani, Nigeria, Risk Factors, Severe Diarrhea

## Abstract

**Purpose:**

The aim of this study was to determine and investigate the risks associated with cataract in South Western and North Central Nigeria.

**Materials and Methods:**

A hospital-based, case–control study was conducted in Lagos (Lagos group), South Western Nigeria, and Kano (Kano group), North Central Nigeria. In this study, 530 subjects with visually impairing cataracts (study group) and 530 age^-^ and sex-matched controls (control group) were recruited from patients aged 40 to 89 years attending the ophthalmology clinics at the same hospital. All subjects were examined for the presence/absence of cataract and interviewed about their educational achievements, diarrhea/dehydration crises, urban/rural residence, and ophthalmological conditions. A standardized questionnaire was administered to all subjects. Logistic regression analysis with age adjustment, literacy, outdoor work, body mass index, crowding, regular vegetable intake, heavy alcohol, and cigarette intake was performed. *P* < 0.05 was considered statistically significant.

**Results:**

Using multivariate regression analysis, after adjustment for age and other demographics factors, low education and no education [adjusted odds ratios (OR) = 2.42 for the Lagos group and 4.10 for Kano group] and a positive history of diarrhea or dehydration crises (adjusted OR = 1.31 for the Lagos group and 2.12 for Kano group) were associated with an increased risk for cataract. Senile cataracts were more common among the Fulani ethnic group (adjusted OR = 2.21) of North Central Nigeria. However, rural or urban residence did not reveal any positive risk for cataract.

**Conclusion:**

The risk of cataract in North Central Nigeria is similar to that in South Western Nigeria. Cataracts were strongly associated with increasing age, with peak age of 55 years and were more common in those with lower education, severe diarrhea and among the members of Fulani in North Central Nigeria.

## INTRODUCTION

Cataract is the most common cause of blindness and visual impairment in Nigeria.^1^ Currently, cataract surgery comprises 60% of all surgeries in Nigeria.^2^ Recent studies by multiple investigators in different regions of Nigeria have identified some major risk factors for cataract.^3^–^5^ These previously published reports may be prone to confounding variables and other limitations that have precluded establishment of a definitive causal relationship.

Lagos and Kano are major commercial cities in Nigeria with population of 9.8 and 9.4 million people, respectively. The people of Lagos are mainly Yoruba speaking while the people of Kano are mainly Hausa and Fulani speaking. Most of the residents in both cities are traders and civil servants while those in surrounding districts (villages) are mainly farmers. Hence, there are ethnic, socioeconomic, cultural, and environmental differences in the rural and urban populations. These two populations serve as representative populations of their respective regions to determine risks factors for cataract. The aim of the present study was to evaluate and determine the risks associated with age-related cataract using case–control studies of two populations in Nigeria.

## MATERIALS AND METHODS

This hospital based case–control study was conducted in a major hospital in Lagos (Lagos group), South Western Nigeria and Kano (Kano group), North Central Nigeria. Both hospitals are tertiary Eye Health Centers that provide standard eye care services and referral for intraocular surgery for both the urban and rural populations of the cities and surrounding villages. All subjects were patients attending the ophthalmology clinics. Subjects with congenital cataracts and acquired cataracts were excluded from the study. The term “acquired cataract” was defined as opacity, partial or complete, of one or both eyes, on or in the lens or capsule for which a specific cause is known (excluding congenital cataract) causing impaired vision or blindness.^6^

The criterion for diagnosis of a cataract for this study was with “sufficiently advanced lens opacity that impaired vision” (study group). While controls were defined as subjects who did not have cataracts (clear lens, no opacity) and were comparable in age and sex (control group). Study subjects were recruited after an ophthalmologist confirmed the diagnosis of a cataract. Subjects in the control group were recruited from the Refractive Error Unit of the same hospital after confirmation of the absence of cataracts. Controls were recruited from the same eye hospital, because there were no comparable hospitals in the geographic areas under study and recruitment from nonhospital facilities was unreliable and extremely difficult.

Informed consent was obtained from all subjects in the study. All subjects underwent a complete examination by senior ophthalmic surgeons that included measurement of visual acuity, manifest refraction, slit lamp examination for central lens opacity, and other tests consistent with the routine clinical protocol.

Personal interviews were conducted with all subjects while they were still in the hospital and immediately after diagnosis. The interview procedures and questionnaire used in this study has been previously tested and standardized in an extensive pilot study of over 1000 interviews at various locations.^7^ From this questionnaire, 80 items were coded in a form suitable for computer database entry to facilitate data analyses. Data were collected on age, sex, tribe, place of residence, social amenities, educational achievements, and ophthalmic details. Urban–rural differences and place of residence (domicile type) was determined according to the methods proposed by Goldestein^8^ as follows: A rural area was defined as an area or community without electricity, industries, and/or other minor social amenities, while urban was classified as cities or communities with electricity, industries and/or other social amenities including pipe borne water. Educational levels were determined by history taking to ascertain the highest level of school grade or higher education completed. For analysis, these levels were grouped into four categories: 0, none; 1, primary (1–6 years); 2, secondary (6–11 years); and 3, post secondary (>11 years). We investigated and collected other demographic factors including heavy alcohol consumption (reference: nonconsumers), heavy cigarette smoking (reference: never smokes), working outdoors (reference: <2 h/day), body mass index (weight in kilograms divided by the square of height in meters) (reference: >17.0 BMI), crowding (reference: ≤3 people/room) and regular vegetable intake (reference: yes). The subjects were asked to accurately estimate the quantity of liquor or beer consumed in the last 10 years. Heavy alcohol consumption was categorized as three to five bottles per day for 10 years. Smoking history was categorized in terms of “never smokers” and “heavy smokers” (5–10 cigarettes/day for 20 years).

Once an interview had been conducted with a subject in the study group, the next subject was recruited as a control from the refractive error unit. The study and control groups were interviewed in exactly the same manner. All subjects were asked if they had severe diarrhea lasting 4 or more days, the time since the last attack and the treatment of the episode of diarrhea. The term severe diarrhea was defined as a history of life-threatening cholera–diarrhea disease, severe enough to warrant bed rest for 4 days or longer.

### Data analyses

Data were recorded on predesigned forms transferable to a computer database. Data were analyzed by multivariate logistic regression analyses using Statistical Package for the Social Sciences (SPSS/PC^+^, SPSS Inc., IL., USA). Two known potential confounders (age and sex) were used as matching variables. In order to assess how a single factor affects the outcome of cataract formation ignoring potential covariates, a crude model of logistic regression was performed. The primary and final analyses incorporated these covariates including age (matching variable) in a multivariate logistic regression model to evaluate the relation between area of residence, ethnic differences, severe diarrhea, and level of educational attainment on the risk of cataract. We considered a variable to be a potential confounder if the difference between the unadjusted and adjusted odds ratios (ORs) were greater than 10% of the unadjusted value.

The data entered into the database consisted of 11,358 value labels accumulated across all variables, and memories allowed for 7764 cells with two dimensions for general cross tabs. ORs proposed by Mantel and Haenszel^9^ were calculated from contingency tables and were used as valid summary estimates of relative risk. Thus, a reported odd ratio of 3.0 for a particular risk factor suggests that the exposed group is three times more likely to have cataract than those not exposed to that factor. Ninety-five percent (95%) confidence intervals as described by Fleiss^10^ were calculated for each factor. A *P* value less than 0.05 was considered statistically significant.

## RESULTS

A total of 1060 subjects aged 40–89 years were interviewed and examined over a 24-month period (January 2006–December, 2007) [Tables 1A and 1B]. Ophthalmic examination identified 530 subjects with cataract and 530 controls. There were 37.9% subjects with unilateral cataract and 62.1% with bilateral cataracts. In Kano, 330 subjects with cataract and an equal number of controls were interviewed and in Lagos 200 subjects with cataract and an equal number of controls were interviewed. The participation rate was 92%. In the study group, 62.7% of the subjects cataract were in the fifth decade of life and 62.5% of the subjects were in the sixth decade of life.

**Table 1A:** Age and sex distribution of the study group and control group from Lagos, Nigeria

Sample group	Age group (years)					Total (%)	Total number of subjects
	40–49	50–59	60–69	70–79	80–89		
Study group^a^							
Males (*n*), (%)	18 (9.0)	49 (24.4)	34 (17.0)	26 (13.1)	5 (2.4)	132 (66.0)	200
Female (*n*), (%)	15 (7.5)	22 (10.8)	20 (10.5)	9 (4.4)	2 (0.75)	68 (34.0)	
Control group^b^							
Male (*n*), (%)	17 (8.3)	56 (27.8)	32 (16.0)	18 (9.3)	3 (1.5)	126 (62.9)	200
Female (*n*), (%)	16 (7.8)	19 (9.3)	21 (10.7)	16 (7.8)	2 (1.0)	74 (37.1)	

a Study group comprises of subjects with visually impairing senile cataract

b Control group comprises sex- and age-matched subjects with no lens opacities (no cataracts)

**Table 1B:** Age and sex distribution of the study group and control group from Kano, Nigeria

Sample group	Age group (years)					Total (%)	Total number of subjects
	40–49	50–59	60–69	70–79	80–89		
Study group^a^							
Males (*n*), (%)	33 (10.0)	82 (24.8)	60 (18.2)	43 (13.0)	10 (3.0)	228 (69.0)	330
Female (*n*), (%)	22 (6.8)	33 (9.9)	32 (9.6)	13 (4.0)	2 (0.70)	102 (31.0)	
Control group^b^							
Male (*n*), (%)	29 (8.9)	81 (24.4)	50 (15.2)	30 (9.2)	8 (2.3)	198 (60.0)	330
Female (*n*), (%)	24 (7.1)	41 (12.4)	39 (11.7)	24 (7.4)	4 (1.2)	132 (40.0)	

a Study group comprises of subjects with visually impairing senile cataract

b Control group comprises sex- and age-matched subjects with no lens opacities (no cataracts)

Descriptive details of the study group, control group, and distribution of ORs for the confounding variables are presented in Tables 2A and 2B. Univariate analysis shows the association of cataract with heavy alcohol and cigarette consumption, working outdoors, crowding, regular vegetable intake, and BMI. These variables were subsequently evaluated as potential confounders.

**Table 2A:** Exposures to different potential confounding variables and univariate associations with cataract in Lagos, Nigeria

Potential confounding variable	Study group^a^ (*n* = 200)	Control group^b^ (*n* = 200)	OR (95% CI)
Work outside (reference: < 2 h/day)	35 (17.5%)	21 (10.5%)	1.80 (1.49–2.89)
Body mass index (reference: >17.0 BMI)	25 (12.5%)	9 (4.5%)	3.03 (2.2–4.62)
Heavy alcohol consumption	36 (18.0%)	20 (10.0%)	2.42 (1.93–3.91)
Heavy cigarette smoking (reference: none)	14 (7.0%)	4 (2.0%)	3.69 (2.07–6.62)
Crowding (reference: ≤3 people/room)	18 (9.0)	32 (16.0)	0.52 (0.32–0.72)
Regular vegetable intake (reference: yes)	41 (20.5%)	38 (19.0%)	1.10 (1.02–2.5)

a Study group comprises of subjects with visually impairing senile cataract

b Control group comprises sex and age matched subjects with no lens opacities (no cataracts), OR: Odds ratio, CI: Confidence interval

**Table 2B:** Exposures to different potential confounding variables and univariate associations with cataract in Kano, Nigeria

Potential confounding variable	Study group^a^ (*n* = 200)	Control group^b^ (*n* = 200)	OR (95% CI)
Work outside (reference < 2 h/day)	69 (21.0%)	27 (8.2%)	5.9 (4.82–6.98)
Body mass index (reference: >17.0 BMI)	38 (11.5%)	18 (5.4%)	2.26 (1.82–3.70)
Heavy alcohol consumption	56 (17.0%)	35 (10.6%)	1.7 (1.48–2.82)
Heavy cigarette smoking (reference: none)	32 (9.7%)	12 (3.6%)	2.84 (2.17–3.51)
Crowding (reference: ≤3 people/room)	27 (8.2%)	23 (6.7%)	1.19 (1.05–1.94)
Regular vegetable intake (reference: yes)	56 (17.0)	39 (11.8%)	1.52 (1.33-2.71)

a Study group comprises of subjects with visually impairing senile cataract

b Control group comprises sex and age matched subjects with no lens opacities (no cataracts), OR: Odds ratio, CI: Confidence interval

Heavy alcohol, cigarette consumption, working outdoors, and BMI were confounders of the relationship among area of residence, ethnic differences, literacy, severe diarrhea, and cataract according to our definition of 10% difference between adjusted and unadjusted value. These were included in the final logistic regression model.

Analysis of the data indicated that there was no association between urban–rural residence and risk of cataract that impairs vision. In Lagos, the ratios of rural-to-urban dwellers were 1:7 in the study group and 1:5 in the control group [adjusted OR = 0.65, 95% confidence limit (CI): 0.39–0.93, *P* = 0.068] [Table 3]. In Kano, the ratio of rural-to-urban dwellers was 4:1 in both the study group and control group (adjusted OR = 0.98, 95% CI: 0.96–1.0, *P* = 0.072) [Table 3].

**Table 3:** Relationship between rural–urban residence and cataract

	Lagos (South Western Nigeria)				Kano (North Central Nigeria)			
	Study group,^a^*N* = 200	Control group,^b^*N* = 200	OR (CI)	*P* value	Study group,^a^*N* = 330	Control group,^b^*N* = 330	OR (CI)	*P* value
Rural	25 (12.5)	36 (18.0%)	0.65 (0.39–0.93)	0.068	268 (81.2%)	269 (81.5%)	0.98 (0.96–1.0)	0.072
Urban	175 (87.5)	164 (82.0%)			62 (18.8%)	61 (18.5%)		
Ratio	1:7	1:5			4:1	4:1		

a Study group comprises of subjects with visually impairing senile cataract

b Control group comprises sex- and age-matched subjects with no lens opacities (no cataracts), OR: Odds ratio, CI: Confidence interval

Among all the ethnic groups (Ibo, Yoruba, Hausa, Fulani, and Edo) in both the Lagos and Kano groups, only members of the Fulani in North Central Nigeria showed a significant association with cataract [Tables 4A and 4B]. There was an approximate doubling of risk (OR = 2.21, 95% CI: 1.81–3.76, *P* = 0.003) for cataract in the Fulani compared to other ethnic groups [Table 4B]. A high yet nonsignificant prevalence of cataract (47.9%) was found among the Hausa speaking people of North Central Nigeria. There were no differences among the ethnic groups in Lagos [Table 4A].

**Table 4A:** Distribution of odds ratios and 95% confidence interval for variables in a study of cataract risk factors in Lagos, Nigeria

	Study group^a^ (*n* = 200)	Control group^b^ (*n* = 200)	Adjusted odds ratio (95% CI)	*P* value
Ethnic group				
Ibo	42 (21.0%)	29 (1.5%)	1.40 (1.06–2.61)	0.068
Yoruba	133 (66.5%)	147 (73.5%)	0.62 (0.46–2.16)	0.082
Hausa	6 (3.2%)	8 (4.0%)	0.67 (0.52–0.93)	0.092
Fulani	3 (1.5%)	4 (2.0%)	0.72 (0.32–1.10)	0.074
Edo	5 (2.5%)	6 (3.0%)	0.79 (0.65–1.72)	0.072
Levels of educational attainment				
Post-secondary (>11 years)	15 (7.5%)	21 (10.5%)	Baseline	
Secondary (6–11 years)	54 (27.0%)	104 (52.0%)	0.61 (1.2–4.33)	0.06
Primary (1–6 years)	64 (32.0%)	42 (21%)	1.74 (2.03–4.92)	0.02
Primary–post secondary (all)	167 (83.5%)	133 (66.5%)	1.69 (1.01–3.03)	0.005
None (illiterate)	67 (33.5%)	33 (16.5%)	2.42 (2.28–5.49%)	0.025
Diarrhea				
None	178 (89.0%)	185 (92.5%)	Baseline	
Severe diarrhea	22 (11.0%)	15 (7.5%)	1.31 (1.02–3.07)	0.02

a Study group comprises of subjects with visually impairing senile cataract

b Control group comprises sex- and age-matched subjects with no lens opacities (no cataracts), CI: Confidence interval

**Table 4B:** Distribution of odds ratios and 95% confidence interval for variables in a study of cataract risk factors in Kano, Nigeria

	Study group^a^ (*n* = 330)	Control group^b^ (*n* = 330)	Odds ratio (95% CI)	*P* value
Ethnic group				
Ibo	11 (3.3%)	16 (4.8%)	0.48 (0.34–0.85)	0.082
Yoruba	20 (6.1%)	19 (5.8%)	0.98 (0.0.62–1.93)	0.088
Hausa	158 (47.9%)	211 (63.9%)	0.48 (0.21–0.93)	0.062
Fulani	118 (35.7%)	58 (17.6%)	2.21 (1.81–3.76)	0.003
Edo	4 (1.2%)	3 (0.8%)	1.10 (0.42–3.62)	0.083
*Levels of educational attainment*				
Post-secondary (>11 years)	6 (1.8%)	23 (6.97%)	Baseline	
Secondary (6–11 years)	17 (5.2%)	34 (10.3%)	1.52 (1.10–3.56)	0.068
Primary (1–6 years)	40 (12.1%)	35 (10.6%)	3.96 (2.82 –6.37)	0.004
Primary–post secondary (all)	63 (19.1%)	92 (27.9%)	2.28 (1.11–4.29)	0.001
None (illiterate)	267 (80.9%)	238 (72.1%)	4.10 (2.46–10.5)	0.001
Severe diarrhea				
None	272 (82.4%)	304 (92.1%)	Baseline	
Severe diarrhea	58 (17.6%)	26 (7.9%)	2.12 (1.09–5.74)	0.02

a Study group comprises of subjects with visually impairing senile cataract

b Control group comprises sex- and age-matched subjects with no lens opacities (no cataracts), OR: Odds ratio, CI: Confidence interval

Lower educational achievement was a strong risk factor for cataract. There was an increasing level of cataract with decreasing levels of educational attainment. When noneducated and educated at different levels were compared, the adjusted ORs were 0.61, 1.74, and 2.42 or 1.52, 3.96, and 4.10 for secondary school, primary school, and non-school attainment in the Lagos group and Kano group, respectively [Tables 4A and 4B]. These values were statistically significant at primary school to noneducational attainment [Tables 4A and 4B] (*P* > 0.05). Multivariate analysis followed by comparison of noneducated (illiterate) to the educated (literate) subjects with logistic analysis while controlling for smoking habits, alcohol consumption, and other variables revealed a significant association between noneducational status and cataract (adjusted OR = 1.69, 95% CI, *P* < 0.005) for the Lagos group and adjusted OR = 2.28, 95% CI, *P* < 0.001 for the Kano group, respectively.

Severe diarrhea appeared as a strong risk factor for cataract for the Lagos and Kano groups. The ORs and 95% CI for severe diarrhea in both Lagos and Kano groups were 1.31 (1.02–3.07) and 2.12 (1.09–5.74), respectively [Tables 4A and 4B]. Analysis of the result suggests a positive and close association between exposure to dehydrational crises as a history of episodes and duration of severe diarrhea and risk of lens opacities that impair vision. Cataract is on the average 1.3 times higher in the Lagos group or 2 times higher in the Kano group in those had a history of severe diarrhea compared to those who did not, which was statistically significant (*P* < 0.02).

## DISCUSSION

Cataract formation is a common response of the lens to a variety of socioeconomic and environmental insults. This study identified three important risk factors for age-related (senile) cataract after adjustment for multiple potential confounding variables in two populations in South Western (Lagos) and North Central (Kano) Nigeria. Logistic regression and multivariate adjustment found the urban-rural residence was not significant in both the Lagos and Kano groups. This observation suggests no difference in place of residence as a potential risk factor for cataract. These findings may indicate that being born and living in rural areas yield no benefit on the risk of cataract and other risk factors such as socioeconomic factors come into play. However, earlier case–control studies have reported an increased risk of cataract in the rural populations of Central Myanmar, Australia and Sumatra, Indonesia.^11^–^13^

Among all the ethnic groups that reside in Lagos and Kano, only members of the Fulani of North Central Nigeria showed a significant association with cataract. This is the first study to investigate the relative risk of cataract associated with ethnicity in Nigeria. The reason for the association between cataract and Fulani remains unclear. The high prevalence of cataract among the Fulani could be attributed to environmental factors, genetics, or both. Analysis of the data revealed that the majority of Fulani of North Central Nigeria rear cattle (Nomadic Fulanis), an occupation which was previously reported (*unpublished results*) as a significant risk factor for cataract. Similar work conducted on the Mangu and the Normadic Fulanis in Plateau State of Northern Nigeria reported an increased risk of cataract.^14^ Similarly, a population-based study in North Central Nigeria has implicated poor sanitation, lower education, socioeconomic factors, and nonprofessional occupations as risk factors for cataract.^15^ In addition, the Fulanis have an adult diet-rich in high lactose milk and milk products. A geographical approach^16^,^17^ to age-related cataract reported that populations with a history of high milk intake and milk product intake in Northern Europe, North West India, North Central Nigeria (Hausa and Fulani), and Arabia appear to have a greater likelihood of developing senile cataract due to a mutation that causes high lactase activity and the accumulation of galactitol in the lens.

We found no evidence to suggest that history of regular vegetable intake altered the incidence of cataract in the Fulani. Perhaps, the occupational hazards of greater ultraviolet light exposure due to exposure to sunlight may account for the increased risk of cataract among the Fulanis of North Central Nigeria. However, there are a number of other factors which also may affect cataract development such as that the inter-relation among diarrhea, nutrition, socioeconomic status including lower educational levels that may affect these outcomes. However, we did find a high prevalence of cataract (though not statistically significant) among the Hausa speaking people of North Central Nigeria. Hausa are the main inhabitants of North Central Nigeria, and this high prevalence may suggest that environmental factors such as ultraviolet exposure and lifestyle rather than genetic differences play a major role in the etiology of cataract-related blindness.

In this study, illiteracy was significantly associated with cataract. For example, there was a high prevalence of cataracts in subjects who could not read or write compared with those who have received western education. Additionally, the longer the period of education, the lower was the risk of cataract. As with many diseases, low socioeconomic status related to educational achievement whether measured by income, education, or occupation is a contributory factor to cataractogenesis. Improvement in economic and educational status may have a positive impact on cataract prevention. The association of cataract with low educational status in the populations studied in this study likely means that these are markers for other risk factors. Previous case–control studies^18^,^19^ have also revealed an association between cataract and educational attainment.

The risk of cataract in North Central Nigeria is similar to that in South Western Nigeria. However, low educational attainment and poor health educational awareness appear to have contributed significantly to the increased risk of cataract in North Central Nigeria compared to that in South Western Nigeria. Therefore given the western education and proper eye care services and awareness of the regional population, a significant proportion of cataract blindness can be prevented.

A history of severe diarrhea has been identified as a major risk factor for cataract from studies by multiple investigations in many developing countries.^20^,^21^ Our outcomes concur with previous published studies as we found a strong association between severe diarrhea and cataract. After controlling for age and other demographics factors including alcohol and cigarette consumption and outdoor work, people who had dehydrational crises had higher risk for cataract by 1.31 times in the Lagos group or 2.12 times in the Kano group. The OR approximately doubled in the study in North Central Nigeria where severe diarrhea was both common and occurred with greater severity.^15^ These findings combined with our results support the hypothesis of a causal association between repeat episodes of dehydrational crises and blindness due to cataract. The causal association was strengthened by the similarity of findings in both study populations, particularly in view of the significant differences in the environmental and sociocultural characteristics of the two populations studied. In contrast, a case-controlled study conducted in the state of Tamil Nadu, Southern India showed and an observational study in Bangladesh found no association between dehydrational crises and cataract.^22^,^23^ Previous researchers have postulated that severe diarrhea can cause cataract by the cyanate-induced carbamylation of lens proteins, osmotic stress, acidosis, and malnutritional crisis.^24^,^25^ For these studies, it was proposed that diarrhea may partially account for the high prevalence of cataract in tropical countries.^24^,^25^

Case–control studies are prone to selection and information bias. Recall bias could be one such factor in this study. The onset of age-related cataract is probably influenced by a number of variables. However, in these studies as in any other case–control study, it is possible that the reported relative risks were grossly over- or underestimated due to bias arising from the study and/or confounding variables. To avoid selection bias, we used the same number of age- and sex-matched controls to minimize differences between the study group and the control group. This increased the possibility that the data reported here more indicative of cataract-related risks. Some of the subjects in the control group who had lens opacities were excluded from the analysis. Failure to exclude subjects in the control group with lens opacities would result in a type 1 error (false positives) causing considerable underestimation of the ORs which would have made identification of risk factors tenous at best. Hence, we believe that the outcomes in this study provide reasonable estimates of ORs that are not exaggerated and should be consider the mathematical minima.

Findings of this study suggest an increased risk of visually disabling cataract in individuals with a positive history of severe diarrhea, illiterates, or individuals with a lower education and among the members of the Fulani tribe of North Central Nigeria. Many of the identified risk factors for cataract in these populations have the potential for being modified through public health interventions. There is, therefore, a need for introduction of interventional measures aimed at reducing exposures to these risk factors.
